# Automating hESC differentiation with 3D printing and legacy liquid handling solutions

**DOI:** 10.1016/j.mex.2016.10.005

**Published:** 2016-10-29

**Authors:** Eric Zluhan, Kathleen Kelly, Nick LeClair, Danique Wortel, Kelsey Moody

**Affiliations:** a Ichor Therapeutics, Inc., Lafayette, NY, USA; b Department of Neuroscience and Physiology, SUNY Upstate Medical University, Syracuse, NY, USA; c College of Veterinary Medicine, Cornell University, Ithaca, NY, USA

**Keywords:** Automated hESC to monocyte differentiation, Automation, Cell culture, Stem cells, Mononuclear cell, Hematology, 3D printing

## Abstract

Historically, the routine use of laboratory automation solutions has been prohibitively expensive for many laboratories. As legacy hardware has begun to emerge on the secondary market, automation is becoming an increasingly affordable option to augment workflow in virtually any laboratory. To assess the utility of legacy liquid handling in stem cell differentiation, a used liquid handling robot was purchased at auction to automate a stem cell differentiation protocol that gives rise to CD14 + CD45+ mononuclear cells. To maintain sterility, the automated liquid handling robot was housed in a custom constructed HEPA filtered enclosure. A custom cell scraper and a disposable filter box were designed and 3D printed to permit the robot intricate cell culture actions required by the protocol. All files for the 3D printed labware are uploaded and are freely available.

• A used liquid handling robot was used to automate an hESC to monocyte differentiation protocol.
• The robot-performed protocol induced monocytes as effectively as human technicians.
• Custom 3D printed labware was made to permit certain cell culture actions and are uploaded for free access.

## Graphical abstract

**Figure:**
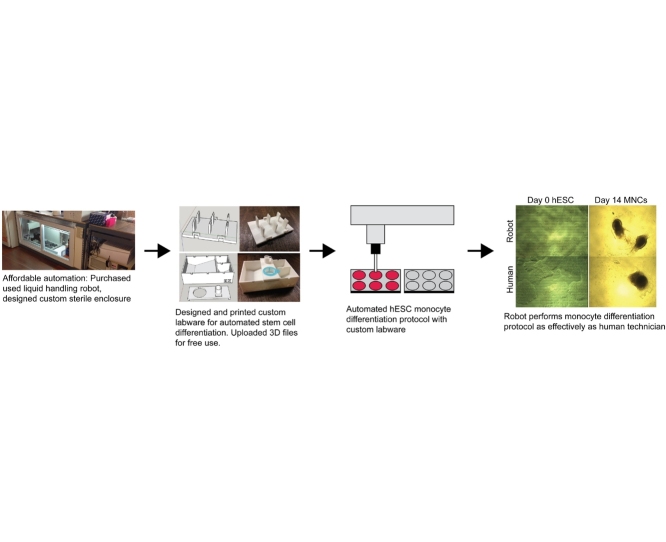

## Methods detail

Here, we assessed the utility of a legacy liquid handling robot at performing a stem cell differentiation protocol that requires intricate and accurate movements. An automated liquid handling robot was purchased used at auction ($100 from eBay.com) and was programmed to perform a stem cell differentiation protocol initially developed by Wilgenburg et al. that gives rise to mononuclear cells [1]. Briefly, the automated liquid handling robot is controlled by a computer and consists of a robotic arm that controls modular pipette attachment tools. Like standard pipettes, the automated liquid handling robot’s pipetting tools are used to transfer liquid from one location to another. The robot also has a gripper tool that can manipulate culture dishes and other lab equipment on the work surface while housed in a custom sterile enclosure. We developed and used custom 3D printed tools to aid the robot in the differentiation protocol and provide the 3D files others to download.

### Modifications to hESC differentiation protocol for use with the liquid handling robot

**Fig. 1:**
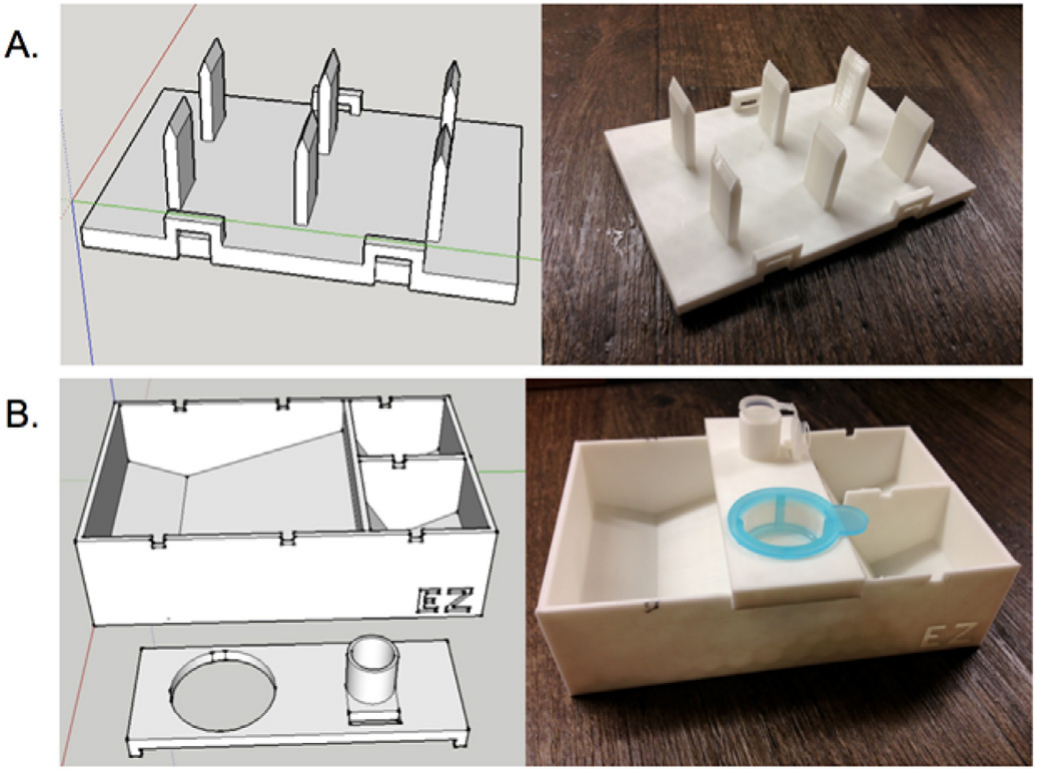
Custom cell scraper and filter box labware. (A) Schematic of cell scraper in 3D modeling computer program (left) and image of 3D printed final product (right). The cell scraper is designed to simultaneously scrape six wells for cell detachment. (B) Schematic of filter box and lid in 3D modeling computer program (left) and image of 3D printed final product (right). The main chamber holds media and cell filtrate. The two small reservoirs hold PBS and Accutase, the micro centrifuge tube holder holds a tube used for obtaining cell concentration.

1. Minor modifications were made to the protocol by Wilgenburg et al. to optimize it for use on the automated liquid handling robot:
  a Wilgenburg et al. plated 20 embryoid bodies from a microplate to a 35 mm well of a 6-well as part of the protocol. However, the multichannel pipette of the automated liquid handling robot has 8 positions, so the robot is instead programmed to transfer 16 embryoid bodies to each well for convenience.
  b Wilgenburg et al. culture hESCs on mouse embryonic fibroblast feeder layers but for this method were maintained on stem cell culture plates with mTeSR-1 since this culture system is more amenable for automation and appears comparable to the co-culture method (data not shown).
2. Custom tools were made to accommodate the robots physical limitations.
  a Trituration combined with enzymatic detachment solution works for detaching loosely adherent cells such as HEK 293 cells, but hESCs strongly adhere to the vessel surface and require a cell scraper. The robot is not equipped with a cell scraper tool, however, its gripper arm can be programmed to readily pick up and manipulate customized pieces of lab equipment. We exploited this functionality and designed and 3D printed a custom cell scraper with 6 positions so that each scraper fit into one well of a 6-well plate for parallel processing (Fig. 1A).
  b We also 3D printed a custom disposable “filter box” that allowed the automated liquid handling robot to filter the cells as described in the protocol by Wilgenburg et al. (Fig. 1B). The filter box was designed to allow the robot’s pipette to dispense cells through a filter and into a main reservoir (155 mL capacity), which could be accessed by the pipette tool to continue the protocol. To utilize as much of the robot’s work surface as possible, the filter box was designed in thirds so that it also contained two small reservoirs for holding phosphate buffered saline (PBS) for washes and Accutase for enzymatic detachment solution (the two chambers each hold ∼35 mL). The filter box also features a holster for a micro centrifuge tube, which was used to collect an aliquot of cells to determine cell concentration. The custom filter box that contained necessary components for the protocol saved significant work surface space. The equipment that would normally occupy 3–4 work surface modules was reduced to 1 module.
  c The cell-scraper and filter box were designed in Google SketchUp**^®^** 3D modeling software. We found it convenient to design custom tools that are based on the dimensions of a typical microplate in the x,y dimensions (∼127.76 mm × 76.49 mm) The schematics were exported as an .stl file and printed on a MakerBot Replicator**^®^** 2 3D printer with white poly lactic acid (PLA) plastic. Standard 3D printed items typically have some degree of porosity and not liquid proof. This can be an issue for continued reuse in sterile conditions. To address this, items can be coated in a waterproof sealant such as epoxy resin or silicone. Also, though we used disposable PLA items here, we have had success treating ABS printed items with acetone, which melds the filament layers and makes the item liquid proof. The 3D printed tools were sterilized by UV light before use and the filter box was discarded after use. The .stl files for these designs are available for download in the Supplementary materials (Table 1).

### Automating the hESC differentiation protocol by Wilgenburg et al.

**Note:** We recommend verifying the robot’s pipetting and cell culture accuracy with an inexpensive cell line such as HEK 293 cells before proceeding with hESC culture (Fig. S1)

**Fig. 2:**
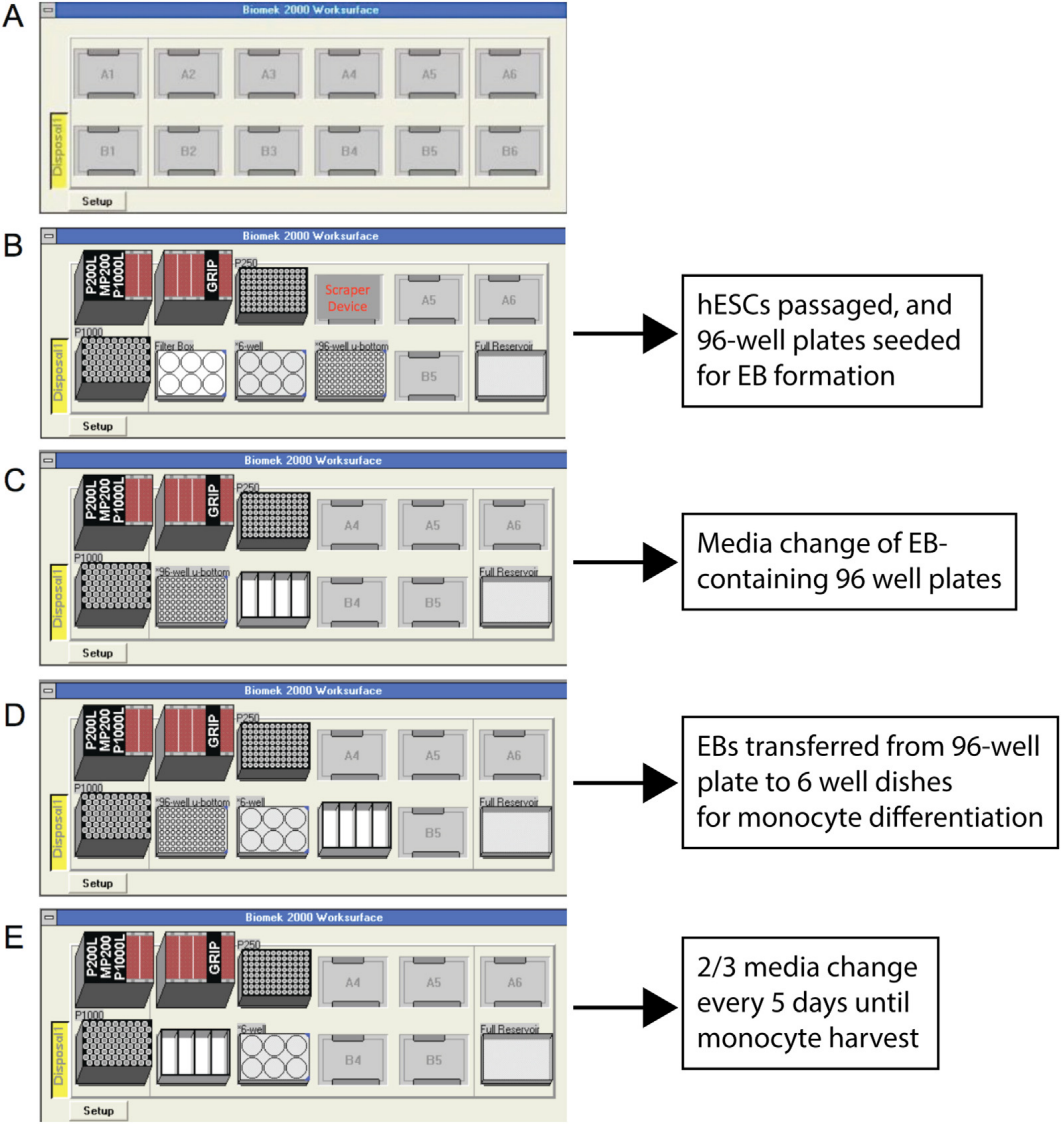
Work surface configurations for automated liquid handling robot. **(A)** Blank deck layout of automated liquid handling robot. The robot comes with 8 deck slots (A2-A5, B2-B5); 2 additional slots were added on each side (A1, A6, B1, B6) to increase work surface area. **(B)** Work surface layout for embryoid body formation step (Day 0). Operator places 6-well and 96-well plates on work surface, 2 mL of PBS and Accutase in small reservoirs, and 5 mL spin media in main reservoir. Custom filter box at B2 is defined as a 6-well plate. A 6-well plate with confluent hESCs and an ultra low adherence round bottom 96-well plate are manually placed on work surface at B3 and B4. **(C)** Work surface layout for 96-well 50uL media change of embryoid bodies (Day 2). Operator places 4.8 mL spin media in reservoir on B3. **(D)** Automated liquid handling robot work surface layout for 96-well to 6-well transfer of embryoid bodies (Day 4). Operator places 96-well (B2) and 6 well plate (B3) on work surface and 13.2 mL of mononuclear cell media in reservoir (B4). **(E)** Automated liquid handling robot work surface layout for 6-well 2/3 media change of mononuclear cells (Day 9). Operator places 6-well plate with mononuclear cells (B3) on work surface and 12 mL monocyte media in reservoir (B2).

**Fig. 3:**
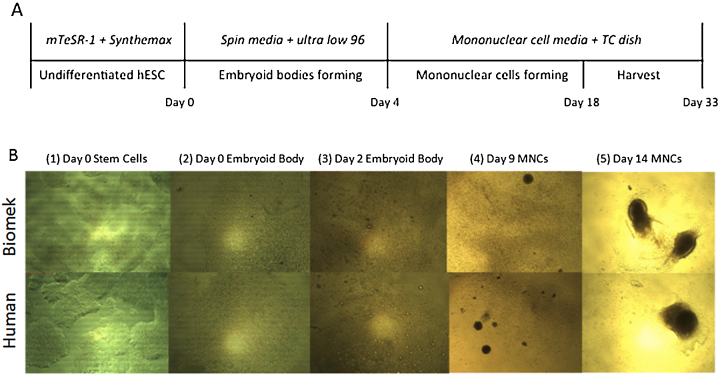
Differentiation of human embryonic stem cells into monocytes. (A) Timeline for hESC differentiation. (B) (1) Undifferentiated hESC before passaging, (2) after being plated and centrifuged in an ultra low attachment 96 well plate, and (3) just prior to transfer to a tissue culture treated 6-well plate. (4,5) Embryoid bodies adhere to culture vessel then give rise to non-adherent monocytes (MNCs).

3 Four work surface configurations were used to support the protocol on the automated liquid handling robot (Fig. 2). Each work surface layout is used for specific step of the differentiation protocol (refer to Fig. 3A).
4 Two wells of hESC (ES-701, BioTime, USA) starter cultures were manually seeded on Synthemax 6-well plates CLS3978, Sigma-Aldrich, USA. When cells were 70–80% confluent, the robot was programmed to passage the cells from 2 to 6 wells. This resulted in 2 new starting wells and 4 wells to be used for the monocyte (MNC) differentiation (Fig. S2). A single well of a 6-well plate yields enough cells for at least two 96-well plates for embryoid body formation. A single 96-well plate yields two 6-well plates for monocyte differentiation with 16 embryoid bodies in each well.
5 Custom movement scripts were programmed in the robot’s software (Bioworks) for non-native movements. These are sequences of simple commands to move the robot arm in the x, y or z direction, manipulate the gripper arm and aspirate or dispense with the pipette. The scripts can be recorded and called via the user interface. Custom movement scripts were particularly useful for using the custom labware. For cell passaging, the robots gripper arm was programmed to pick up the cell scraper and scrape the wells. Then the robot’s pipette tool was programmed to move to four corners of each well for trituration to ensure cell/media removal.

**Note:** Robots should be calibrated before recording scripts and before performing protocols to ensure accurate execution.

a The robot was able to perform all of the steps of the hESC differentiation protocol but was not equipped with a centrifuge for the 96-well embryoid body formation step and required the user to manually centrifuge the plate. However, it is possible to equip the robot with a centrifuge if desired (Table 2).

## Method validation

### Comparison of robot and human technicians performing the hESC differentiation protocol

**Fig. 4:**
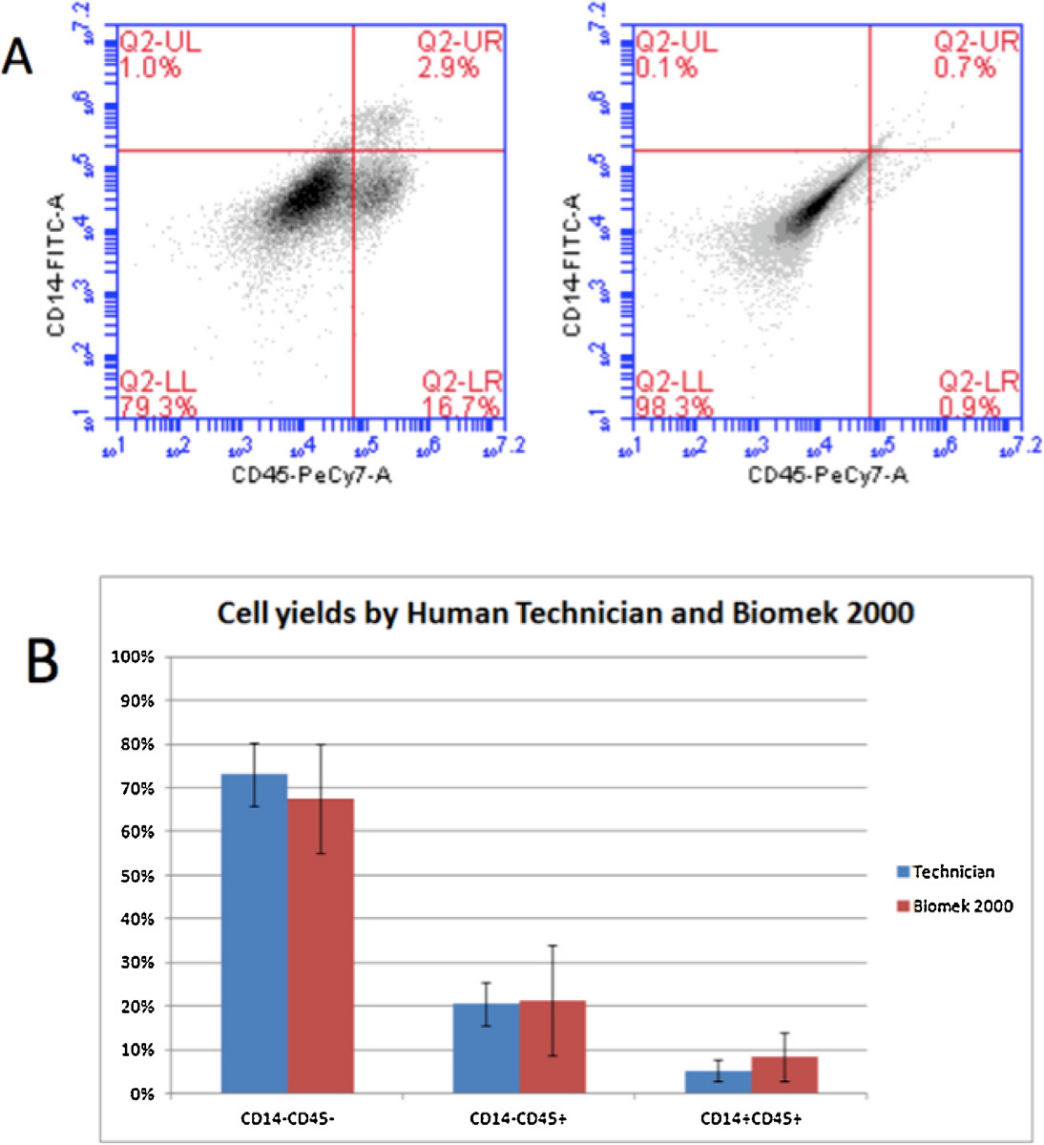
Representative data by flow cytometry for monocyte differentiation. (A) Representative flow cytometry plots for CD14+ CD45+ hESC derived monocytes. Specific antibodies (left) vs. isotype controls (right). (B) Technician and automated liquid handling robot results for hESC-derived monocytes harvested on day 33 are double positive for CD14 and CD45. n = 5 for all and p < 0.43, p < 0.92, and p < 0.29 respectively using student’s two-tailed *t*-test.

The robot and a human technician performed the monocyte differentiation protocol (Fig. 3A) and their performance was compared. The robot-cultured and human-cultured cells and embryoid bodies had similar morphology throughout the differentiation process (Fig. 3B). Cell viability was also compared and was comparable (data not shown). After 33 days in culture, cells in suspension were harvested, treated with anti-CD45 and anti-CD14 and assessed by flow cytometry. Wilgenburg et al. reported an 8.97% CD14 + CD45+ monocyte yield 33 days after embryoid body formation. Here the human technician yielded 5.22% (±2.42%) and the robot yielded 8.38% (±5.69%) CD14 + CD45+ cells per well (Fig. 4). These results are similar to those reported by Wilgenburg et al. CD14-CD45-, CD14-CD45+, and CD14 + CD45+ percent yields were not significantly different between a human technician and the robot (n = 5 for all and p < 0.43, p < 0.92, and p < 0.29, respectively). Taken together, the liquid handling robot is able to perform a complex stem cell differentiation protocol with comparable monocyte yields to human technicians.

## Additional information

Reproducibility is critical when using sensitive stem cell-based assays. Automation provides a solution to side-step user error and standardizes methods. It is important to budget time for a development period before implementing an automated platform into existing protocols. We optimized a number of handling variables including culture conditions, passaging methods and incubation times in order to optimize the protocol by Wilgenburg et al. for the robot’s use. It is important to take care in programming liquid handling robots and in preparing starting reagents to avoid downstream errors. For this protocol in particular, stem cell seeding density and pluripotency is key for success. Calibration and development steps are also critical to obtain accuracy and consistency in automated protocols. Several custom tools were designed for this protocol, but other protocols may have different requirements. For example, 3D parts may require waterproofing for repeated use in sterile conditions. There are also online stores (e.g. shapeways.com) that offer less common printing materials that may be more suited to different uses.

Making custom labware and other files freely available can reduce development phases and increase collaboration between laboratories. Here we have uploaded the 3D printing files for others to download and modify as they see fit. Other labs can reproduce this study or change the files directly to suit their own automation needs. We propose that this type of open-source mentality and use of legacy hardware can shrink certain barriers to entry for budding research and development teams.

## Disclosures

KM holds equity positions in several for-profit stem cell therapy companies, including Ichor Therapeutics, Inc., ImmunePath, Inc., and Advanced Cell Technologies, Inc. EZ and DW hold equity positions in Ichor Therapeutics, Inc. The authors declare no other disclosures.

## Figures and Tables

**Table 1:** Comprehensive list of catalog numbers.

Name	Vendor	SKU	Description
Biomek 2000	Beckman Coulter, Inc.		Legacy liquid handling robot used for autotmation protocols
MakerBot Replicator 2	MakerBot^®^ Industries, LLC		3D printer used to print “scraper box” and “filter box”
Falcon™ Cell Strainers	Fisher Scientific	08−771-1	Used for filter box
mTeSR-1	STEMCELL Technologies	5850	Media for culturing hESC
BMP-4	ProSpec	cyt-081-b	Add to “spin media”
VEGF	Sino Biological	11066HNAB	Add to “spin media”
SCF	Sino Biological	10451-H08B	Add to “spin media”
Rock inhibitor (Y-27632)	Selleckchem	s1049	Add to “spin media”
Antibiotic/antimycotic	Corning	30-004-CL	Add to “spin media and mononuclear cell media
X-VIVO 15	Lonza	04-744Q	Add to “mononuclear cell media”
M-CSF	Sino Biological	11792-HNAH	Add to “mononuclear cell media”
IL-3	Sino Biological	11858-HNAE	Add to “mononuclear cell media”
GlutaGro Supplement	Corning	25-015-CL	Add to “mononuclear cell media”
2-mercaptoethanol	Thermo Scientific	35602	Add to “mononuclear cell media”
Synthemax 6-Well plate	Corning	3978XX1	Used to culture hESC
96-well ultra low attachment plate	Corning	7007	Used for embryoid body formation
Accutase	Innovative Cell Technologies	AT-104	Enzymatic detachment solution used for stem cell detachment
Countess Automated Cell Counter	Invitrogen	C10227	Used for analyzing cell counts
Human Embryonic Stem Cell Line	ESI-035	ES-701	hESC used for protocol
Google SketchUp	Google		Drafting software used to design 3D printed objects
BioWorks	Beckman Coulter		Software controlling Biomek 2000

**Table 2:** Media recipes for hESC differentiation protocol.

Spin media (50 mL)	Mononuclear cell media (50 mL)
49.5 mL mTeSR-1	49.0 mL X-VIVO 15
50 ng/mL BMP-4	100 ng/mL M-CSF
50 ng/mL VEGF	25 ng/mL IL-3
20 ng/mL SCF	55 μM 2-mercaptoethanol
10 mM Y-27632	500 μL-glutamine 100 x liquid
500 μL Antibiotic-antimycotic (10,000 units/mL penicillin G, 10 mg/mL streptomycin sulfate and 25 μg/mL amphotericin B)	500 μL of antibiotic-antimycotic solution (10,000 units/mL penicillin G, 10 mg/mL streptomycin sulfate and 25 μg/mL amphotericin B)
