# Supplementary material for: Automating hESC differentiation with 3D printing and legacy liquid handling solutions
Source: MethodsX. 2016 Oct 29;3:569–76. doi: 10.1016/j.mex.2016.10.005 (PMC5109851; doi:10.1016/j.mex.2016.10.005)
Supplement: Supplementary file 1 [file mmc1.docx]

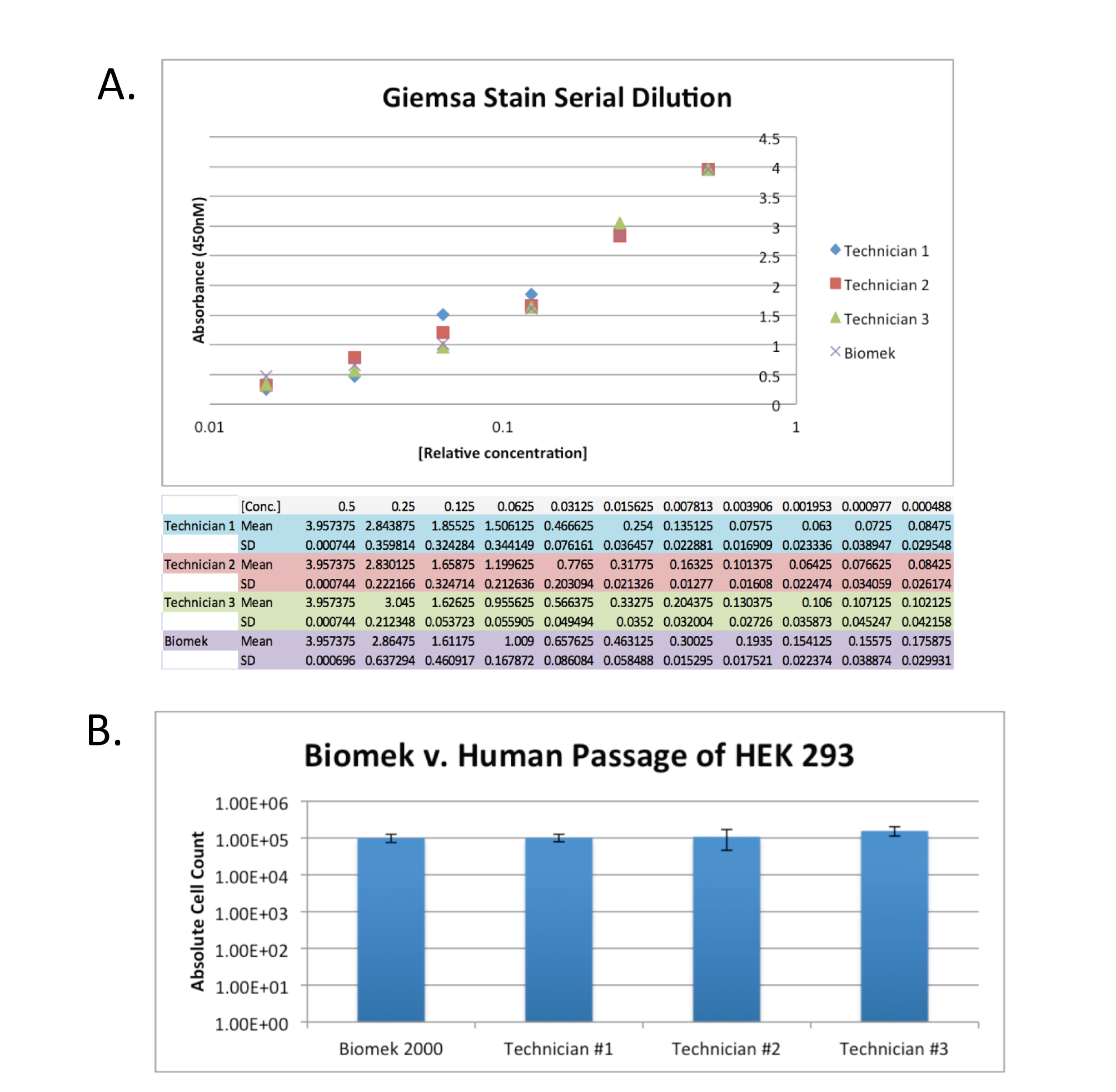


**Figure S1: Automated liquid handling robot performs cell culture with accuracy comparable to human technicians.** (A) To compare pipette accuracy, the robot and human technicians performed serial dilutions of Giemsa stain into water and a microplate reader measured the absorbance of each dilution. Absorbance values were averaged for 8 wells for each concentration measured in the dilution series. Concentration and standard deviation are shown below. (B) The automated liquid handling robot demonstrated comparable cell passaging yields of HEK 293 cells compared to human technicians. Graphical data are the average cell counts ± S.D. of the 6 passaged wells. ANOVA analysis of the passaging efficiency showed there is no statistical significant difference between the three technicians and the biomek p > 0.13.


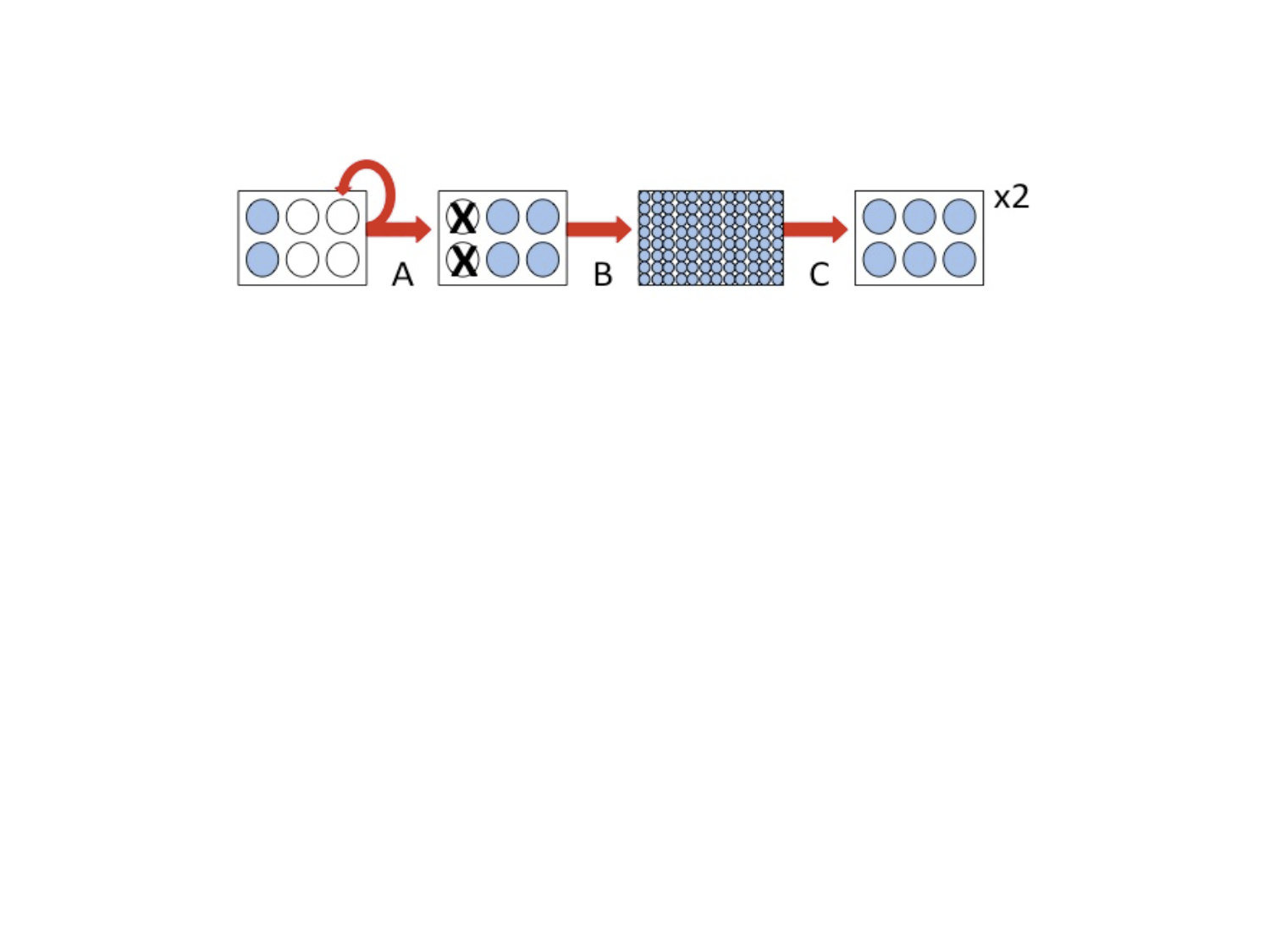


**Figure S2: Culture plate passage diagram.** (A) Stem cell culture plates containing hESC colonies are passaged at a 2:6 ratio. This strategy restores the starting material with each passage, and provides a 6-well plate with 4 wells for use in differentiation. (B) hESC colonies in 4 stem cell culture plate wells are detached, diluted, then plated in an ultra low adherent 96 well plate to form embryoid bodies. (C) 8 embryoid bodies, corresponding to 8 wells of a 96 well plate, are pooled in each 35mm well of a 6-well dish for the final steps of differentiation. Each 96 well plate yields 2 6-well plates.

**Figure S3.** A.) Automated liquid handling robot in custom sterile enclosure. B.) Representative sketch of automated liquid handling robot in sterile enclosure; its components: (1) Biomek 2000 automated liquid handling robot, (2) work surface, (3) main light, (4) UV lights, (5) waste bin, (6) air purifier, (7) control panel for air purifier, (8) lights and automated liquid handling robot on/off switch, (9) duct for air purifier.

Link to .stl files:

<https://www.dropbox.com/sh/yqti0k59akcrwbq/AADWfKm6hQdX6v2dOJcjWTIma?dl=0>
